# Circulating and Urinary CCL20 in Human Kidney Disease

**DOI:** 10.3390/ijms262110563

**Published:** 2025-10-30

**Authors:** Noelia Molina-Cazallas, Diego García-Ayuso, Beatriz Fernández-Fernández, Laura Rodríguez-Osorio, Jinny Sanchez-Rodriguez, María Vanessa Pérez-Gómez, Alberto Ortiz, Adrián M. Ramos

**Affiliations:** 1 Laboratorio de Nefrología e Hipertensión, Instituto de Investigación Sanitaria-Fundación Jiménez Díaz (IIS-FJD), Universidad Autónoma de Madrid (UAM), 28040 Madrid, Spain; noelia.mcazallas@iis-fjd.es (N.M.-C.); diegogarcia2195@gmail.com (D.G.-A.); 2 RICORS2040-Renal, Instituto de Salud Carlos III (ISCIII), 28029 Madrid, Spain; bfernandez@fjd.es (B.F.-F.); jinny.sanchez@iis-fjd.es (J.S.-R.); mvanessa@fjd.es (M.V.P.-G.); 3 Departamento de Nefrología e Hipertensión, Instituto de Investigación Sanitaria-Fundación Jiménez Díaz (IIS-FJD), Universidad Autónoma de Madrid (UAM), 28040 Madrid, Spain; 4 Servicio de Nefrología, Hospital Universitario General de Villalba, 28400 Madrid, Spain; lrodriguezos@quironsalud.es; 5 Departamento de Medicina, Facultad de Medicina, Universidad Autónoma de Madrid (UAM), 28029 Madrid, Spain

**Keywords:** CC motif chemokine ligand 20 (CCL20), chronic kidney disease, diabetic kidney disease, autosomal dominant polycystic kidney disease, clinical outcomes, observational study

## Abstract

CC motif chemokine ligand 20 (CCL20), a chemokine involved in immune cell migration through its receptor CCR6, has been implicated in kidney inflammation in crescentic glomerulonephritis and acute kidney injury. However, clinical information for other kidney diseases is scarce. We have analysed CCL20 levels in plasma and urine from patients with diabetic kidney disease (DKD, *n* = 98) and autosomal dominant polycystic kidney disease (ADPKD, *n* = 85) treated according to the guidelines and studied their association with baseline characteristics and long-term (median follow-up 4.9 and 7.1 years, respectively) clinical outcomes. Single-cell kidney transcriptomics were mined to identify CCL20-expressing cells. Plasma CCL20 was higher in DKD and ADPKD than in a reference group: median 12.8 (3.5–33.2), 6.0 (1.2–19.2), and 0.0 (0.0–9.0) pg/mL, respectively. Urinary CCL20 was quantifiable in 48% of patients with DKD but not in the reference group. Transcriptomics data support a local kidney source of CCL20. In DKD, plasma CCL20 was higher in early compared to advanced CKD. Urinary CCL20 was higher in patients with A2 albuminuria than in those with other albuminuria categories. In ADPKD, higher plasma and urinary CCL20 levels tended to be associated with lower eGFR, higher albuminuria, and larger kidneys. However, no significant association was found between CCL20 levels and progression to kidney failure or death. In conclusion, CCL20 is increased in biological fluids and locally produced in CKD. While this may point to a potential role in risk stratification, further studies are necessary.

## 1. Introduction

Chronic kidney disease (CKD) is among the fastest-growing causes of death worldwide [1]. The most common causes are diseases in the cardiovascular–metabolic–kidney (CKM) spectrum, such as type 2 diabetes mellitus [2]. However, among prevalent patients on kidney replacement therapy, inherited kidney diseases and congenital anomalies of the kidney and urinary tract (CAKUT) are the second most common cause, after glomerulonephritis [3]. Autosomal dominant polycystic kidney disease (ADPKD) is the most common inherited kidney disease [3]. Contrary to the CKM spectrum, kidney disease is the primary event in ADPKD, as it does not occur secondary to systemic alterations such as hyperglycaemia or hypertension [4].

CC motif chemokine ligand 20 (CCL20) is a chemokine expressed in immune cells, such as dendritic cells, macrophages, and T lymphocytes, as well as in tissue-specific cells, including fibroblasts, stromal cells, and epithelial cells of the hepatic, prostatic, and renal tissues [5,6]. CCL20 interacts with a single receptor, the CC chemokine receptor type 6 (CCR6), which is expressed on T helper 17 cells (Th17 cells), regulatory T cells (Treg cells), B lymphocytes, dendritic cells, and neutrophils, regulating their migration during inflammation [7]. The activity of the CCL20/CCR6 axis has been associated with the migration of specific immune cell types and pro-inflammatory activity in experimental models of chronic arthritis, glomerulonephritis, or inflammatory bowel disease [6,8]. Moreover, in kidney transplant recipients, urinary excretion of CCL20 correlated with dendritic cell activity and rejection episodes [9]. In cultured renal tubular cells, stress induced by excessive ambient glucose leads to the expression of CCL20 [10]. CCL20 may activate CCR6 in Th17 cells, promoting inflammation [11,12,13] but also on Treg lymphocytes, dampening inflammation [14]. Ramos et al. demonstrated increased renal and urinary levels of CCL20 in both experimental and human acute kidney injury (AKI) [15]. Surprisingly, functional inhibition of CCL20 using neutralising antibodies or CCR6 deficiency worsened the course of nephrotoxic AKI, increasing mortality and kidney fibrosis [15]. More recent data confirmed CCL20 upregulation in human AKI but observed protection from experimental ischemia–reperfusion AKI to CKD transition upon CCL20 blockade [16].

Overall, there is evidence for both deleterious and protective effects of CCL20 depending on the type and course of the disease. We therefore hypothesised that CCL20 levels in biological fluids could be associated with kidney disease outcomes. The primary aim of this study was to analyse plasma and urinary CCL20 levels in human CKD caused by diabetes or ADPKD, representing examples of kidney disease in response to systemic events and of a primary non-immune kidney disease, respectively. A subsequent primary aim was to assess the association of CCL20 levels with baseline disease severity and long-term kidney and survival outcomes.

## 2. Results

### 2.1. Study Population

A total of 98 patients with diabetic kidney disease (DKD) and 85 patients with ADPKD were included in the analysis (Table 1). The median age for DKD patients was 69.1 years (IQR: 60.4–76.5); 76.5% were male. The median estimated glomerular filtration rate (eGFR) was 57.6 mL/min/1.73 m^2^ (IQR: 70.7–83.2), and the median urinary albumin-to-creatinine ratio (UACR) was 159.1 mg/g (IQR: 36.9–437.7). At baseline, no patients (0%) were on kidney replacement therapy (KRT). During a median follow-up of 4.9 years (IQR: 2.9–5.5), 21 patients (21.4%) died and 4 (4.1%) initiated KRT. The median age for ADPKD patients was 54.7 years (IQR: 43.2–67.5); 45.9% were male. The median eGFR was 59.3 mL/min/1.73 m^2^ (IQR: 37.5–95.6), and the median UACR was 25.3 mg/g (IQR: 7.0–77.3). At baseline, 10 patients (11.8%) were already on KRT. During a median follow-up of 7.1 years (IQR: 2.9–8.4), 28 patients (32.9%) died, and 10 (11.8%) initiated KRT. Cohort distributions by kidney function stage (G1–G5) and albuminuria (A1–A3) are shown in Appendix A Table A1 (DKD) and Table A2 (ADPKD). In the ADPKD group, characteristics are also stratified by the Mayo Clinic TKV classification (Table A2).

The reference group included 11 people without evidence of kidney disease or other medical conditions. The median age was 38 years (IQR: 27–44, *p* < 0.05 vs. CKD), and 54.5% were male (*p* > 0.05 vs. CKD).

### 2.2. Plasma CCL20 in Diabetic Kidney Disease

#### 2.2.1. Assessment of CCL20 Levels in Diabetic Kidney Disease and Relationship with Disease Categories

**Figure 1:**
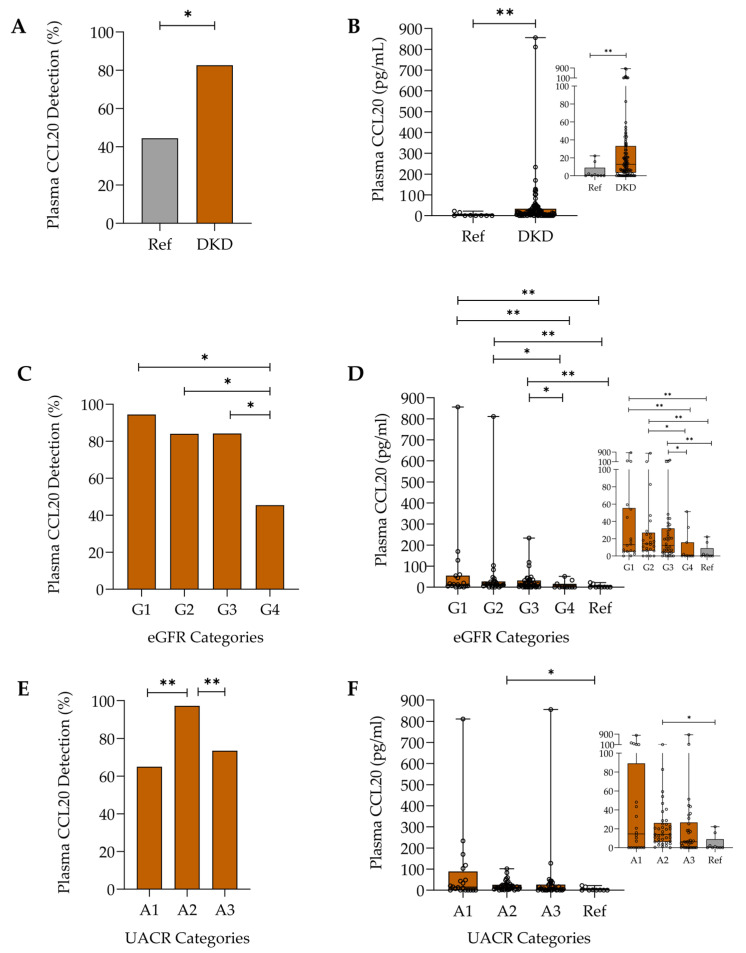
Plasma CCL20 detection and levels in the reference group (Ref) and DKD patients measured by enzyme-linked immunosorbent assay (ELISA): (**A**) percentage of participants with detectable plasma CCL20, * *p* < 0.05; (**B**) plasma CCL20 protein levels. Data are presented as boxplots showing median, interquartile range (IQR), and range, ** *p* < 0.01; (**C**) percentage of detectable plasma CCL20 among DKD patients across different eGFR categories, * *p* < 0.05; (**D**) plasma CCL20 protein levels in DKD patients across different eGFR categories. Data are presented as boxplots showing the median, IQR, and range, * *p* < 0.05, ** *p* < 0.01; (**E**) percentage of detectable plasma CCL20 among DKD patients across different UACR categories, ** *p* < 0.01; (**F**) plasma CCL20 protein levels in DKD patients across different UACR categories. Data are presented as boxplots showing median, IQR, and range, * *p* < 0.05. Inset charts show a magnified view of the lower range of values (**B**,**D**,**F**).

Several studies based on preclinical and translational data predict the involvement of the CCL20/CCR6 axis in the development of diabetes and DKD, the leading cause of CKD [17,18,19]. However, data exploring CCL20 plasma and urine levels in patients with this pathological condition are lacking. Plasma CCL20 was more frequently quantifiable in patients with DKD than in the reference group (82.6% vs. 44.4%, *p* < 0.05) (Figure 1A). Additionally, plasma CCL20 levels were higher in patients with DKD (median and IQR: 12.8 (3.5–33.2) vs. 0.0 (0.0–9.0) pg/mL) (Figure 1B). Surprisingly, both the highest prevalence of quantifiable plasma CCL20 (Figure 1C) and plasma CCL20 levels (Figure 1D) were found in patients with early DKD (better preserved eGFR) and were significantly lower in individuals with more advanced CKD than with early CKD. A similar trend was observed for albuminuria. Patients with A2 albuminuria had the highest prevalence of quantifiable plasma CCL20 (Figure 1E), but plasma CCL20 levels did not significantly differ across albuminuria categories (Figure 1F).

**Figure 2:**
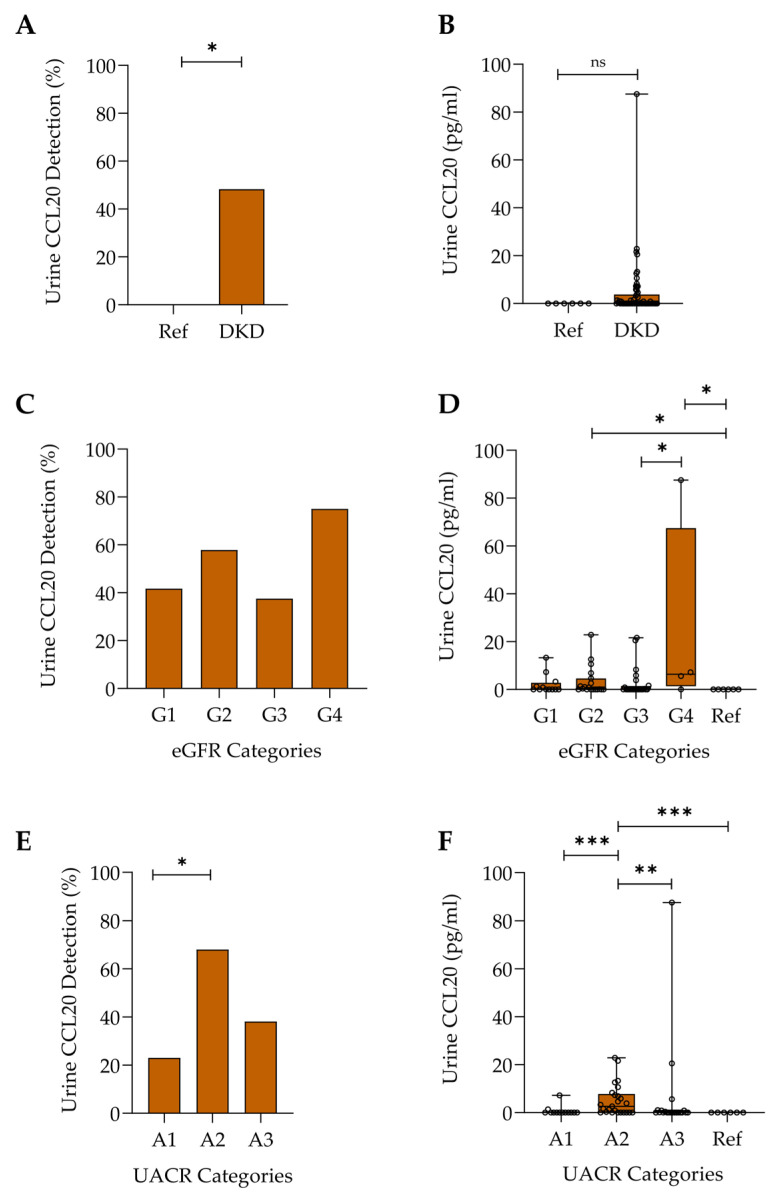
Urine CCL20 detection and levels in the reference group (Ref) and DKD patients detected by ELISA: (**A**) percentage of participants with detectable CCL20 in the urine, * *p* < 0.05; (**B**) urine CCL20 protein levels. Data are presented as boxplots showing the median, interquartile range (IQR), and range. Not significant (ns), *p* = 0.0534; (**C**) percentage of detectable urine CCL20 in DKD patients across different eGFR categories. (**D**) Urine CCL20 protein levels measured by ELISA in DKD patients across different eGFR categories. Data are presented as boxplots showing the median, IQR, and range, * *p* < 0.05; (**E**) percentage of detectable urine CCL20 among DKD patients across different UACR categories, * *p* < 0.05; (**F**) urine CCL20 protein levels measured by ELISA in DKD patients across different UACR categories. Data are presented as boxplots showing the median, IQR, and range, ** *p* < 0.01, *** *p* < 0.001.

We next assessed urinary CCL20 excretion. Urinary CCl20 levels were more often below the limit of detection than the plasma samples. However, similar to plasma CCL20, urine CCL20 was more frequently quantifiable in patients with DKD than in the reference group (48.3% vs. 0.0%; *p* < 0.05) (Figure 2A). Additionally, the highest levels of urine CCL20 were found in some patients with DKD (Figure 2B), although the differences between groups did not reach statistical significance (*p* = 0.0534). There were no significant differences between eGFR categories in the prevalence of quantifiable urinary CCL20 (Figure 2C). However, DKD patients with CKD G4 had the highest urinary CCL20 levels (Figure 2D). As was the case for plasma CCL20, the prevalence of quantifiable urine CCL20 was highest in patients with A2 albuminuria (Figure 2E), who also had the highest urinary CCL20 levels (Figure 2F).

Finally, no differences in the levels of CCL20 between men and women in plasma (13.7 pg/mL male vs. 8.9 pg/mL female; *p* = 0.18) and urine (0.0 pg/mL male vs. 0.6 pg/mL female; *p* = 0.17) were observed.

#### 2.2.2. Relationship Between Plasma and Urinary CCL20 Levels

**Figure 3:**
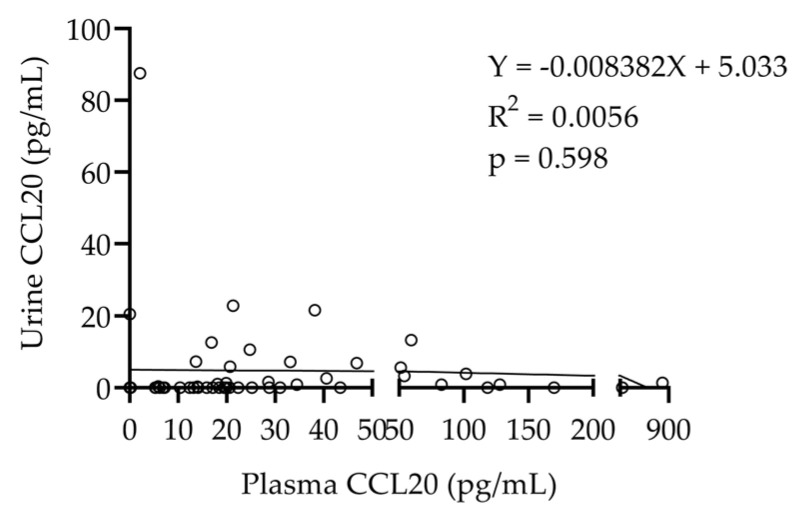
Relationship between plasma and urinary CCL20 levels in DKD patients. A linear regression analysis showed no significant association.

There was no significant relationship between plasma and urinary CCL20 levels, suggesting that higher urinary CCL20 levels are not a result of higher circulating CCL20 levels (Figure 3).

### 2.3. Plasma CCL20 in Autosomal Dominant Polycystic Kidney Disease

**Figure 4:**
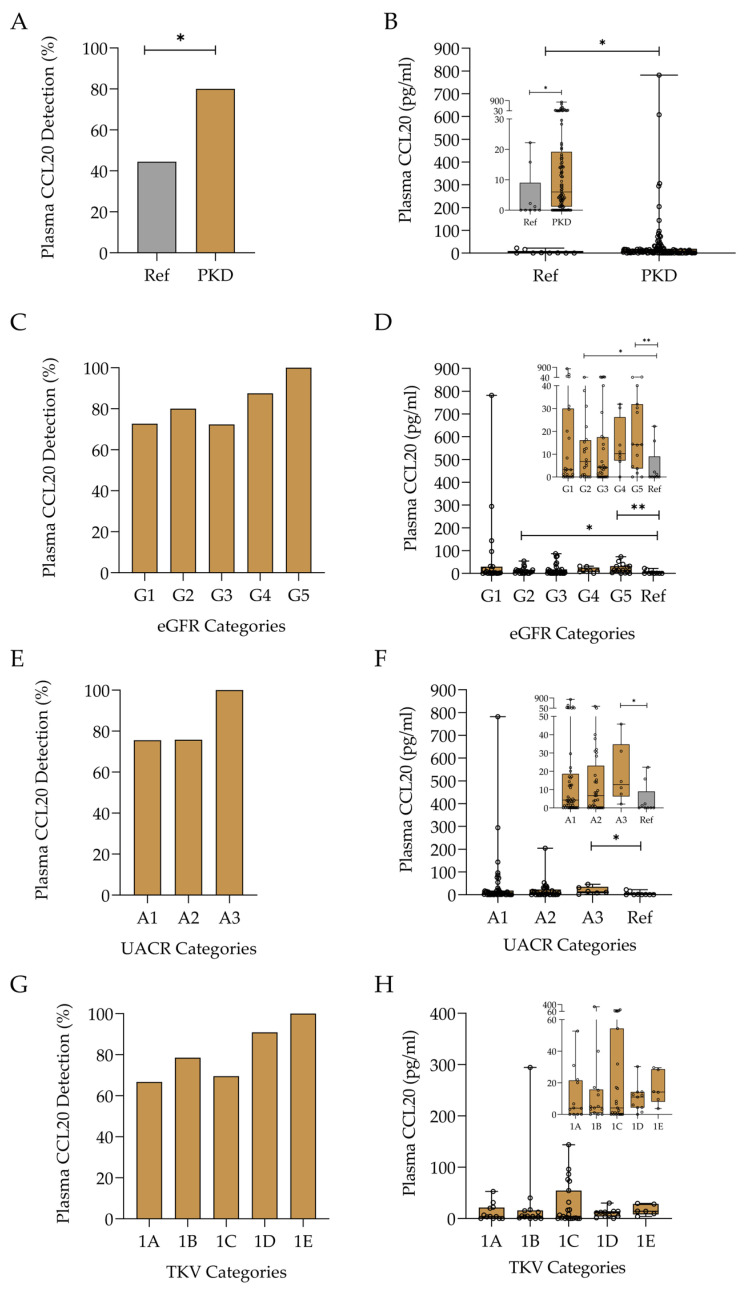
Plasma CCL20 detection and levels in the reference group (Ref) and ADPKD patients assessed by ELISA: (**A**) percentage of participants with detectable plasma CCL20, * *p* < 0.05; (**B**) plasma CCL20 protein levels. Data are presented as boxplots showing the median, interquartile range (IQR), and range, * *p* < 0.05; (**C**) percentage of detectable plasma CCL20 among ADPKD patients across different eGFR categories. (**D**) Plasma CCL20 protein levels in ADPKD patients across different eGFR categories. Data are presented as boxplots showing the median, IQR, and range, * *p* < 0.05, ** *p* < 0.01; (**E**) percentage of detectable plasma CCL20 among ADPKD patients across different UACR categories; (**F**) plasma CCL20 protein levels in ADPKD patients across different UACR categories. Data are presented as boxplots showing the median, IQR, and range, * *p* < 0.05; (**G**) percentage of detectable plasma CCL20 among ADPKD patients across different total kidney volume (TKV) categories; (**H**) plasma CCL20 protein levels in ADPKD patients across different TKV categories. Data are presented as boxplots showing the median, IQR, and range. Inset charts show a magnified view of the lower range of values (**B**,**D**,**F**).

DKD develops secondary to a systemic metabolic defect, characterised mainly by hyperglycaemia and downstream effects such as hyperglycaemia-induced podocyte injury, albuminuria, extracellular matrix production, tubular cell stress, and proinflammatory responses involving innate and adaptive immunity. We next assessed plasma CCL20 in ADPKD, a primary kidney disease that is not immunologically mediated. Furthermore, in ADPKD, albuminuria is a late and mild event, observed in patients with advanced disease, unlike DKD, in which albuminuria frequently precedes the loss of GFR. In this analysis, plasma CCL20 was more often detected in patients with ADPKD than in the reference population (80.0% vs. 44.4%, *p* < 0.05) (Figure 4A). Moreover, plasma CCL20 levels were higher in patients with ADPKD (median and IQR: 6.0 (1.2–19.2) vs. 0.0 (0.0–9.0) pg/mL), *p* < 0.05 (Figure 4B). Although there were no significant differences in the prevalence of quantifiable CCL20 or CCL20 levels among eGFR, albuminuria, or TKV categories, the highest median levels were found in patients with more advanced disease, including those with G5 eGFR, A3 albuminuria, and TKV categories 1D and 1E (Figure 4C–G).

No differences in the levels of CCL20 between men and women in plasma (7.9 pg/mL male vs. 3.3 pg/mL female; *p* = 0.09) were observed.

### 2.4. CCL20 and Progression of Chronic Kidney Disease to Kidney Failure

**Figure 5:**
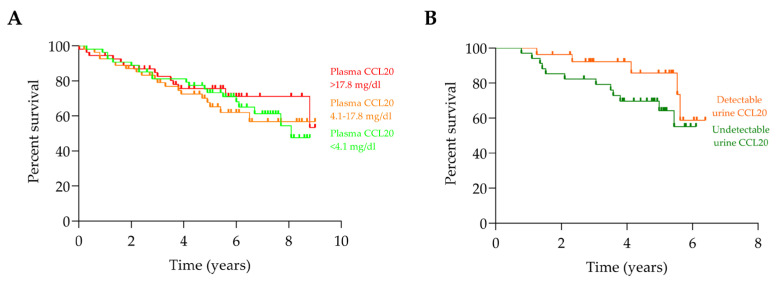
Kaplan–Meier survival curves for freedom from KRT or death. (**A**) Kaplan–Meier survival curves in the combined cohort of DKD and ADPKD patients, stratified by plasma CCL20 tertiles. ADPKD patients already on dialysis at baseline (time 0) were excluded from this analysis. No significant differences were observed among survival curves. Log-rank test: χ^2^ = 0.85, gl = 2, *p* = 0.65. (**B**) Kaplan–Meier survival curves in DKD patients comparing those with detectable versus undetectable urinary CCL20 levels. No significant differences were observed among survival curves. Log-rank test: χ^2^ = 1.77, df = 1, *p* = 0.18. The hazard ratio (HR) was 2.00 (95% CI: 0.77–5.19).

To evaluate the potential impact of CCL20 levels on disease progression, a Kaplan–Meier survival analysis was performed using hard clinical outcomes, including death or initiation of KRT. Since plasma CCL20 levels were generally similar between patients with DKD (82.6% detectable, median 12.8 (3.5–33.2) pg/mL) and ADPKD (80% detectable, median 6.0 (1.2–19.2) pg/mL), these hard outcomes were analysed in both cohorts combined. Additionally, a survival analysis based on urinary CCL20 levels was conducted in DKD patients. The Kaplan–Meier curves showed no significant differences in death or KRT events between plasma CCL20 tertiles in patients with DKD or ADPKD (Figure 5A), nor between patients with detectable or undetectable urinary CCL20 in DKD (Figure 5B).

### 2.5. Identification of Renal CCL20 Sources in Kidney Transcriptomic Databases

**Figure 6:**
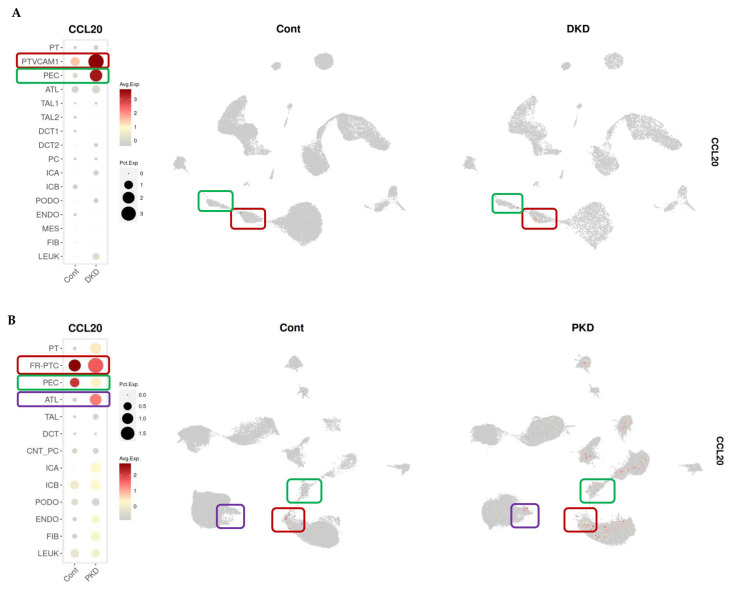
CCL20 expression in kidney cell populations from patients with DKD (**A**) and ADPKD (**B**). Data visualised using the Kidney Interactive Transcriptomics (KIT) platform, developed by the Humphreys Lab (https://humphreyslab.com/SingleCell/) [20], based on single-nucleus RNA sequencing (snRNA-seq) datasets from references [21] and [22], respectively. The dot plots (left) indicate average expression (colour scale) and proportion of expressing cells (dot size) across kidney cell populations. Uniform manifold approximation and projection (UMAPs) (right) illustrate the spatial distribution of CCL20-positive cells in snRNA-seq datasets. Red boxes in the dot plots and UMAPs indicate the cell populations with CCL20 expression. Cont: control; PT: proximal tubule; PTVCAM1: proximal tubular cells expressing VCAM; PEC: parietal epithelial cells; ATL: ascending thin limb of the loop of Henle; TAL1: thick ascending limb 1; TAL2: thick ascending limb 2; DCT1: distal convoluted tubule 1; DCT2: distal convoluted tubule 2; PC: principal cells; ICA: intercalated cells A; ICB: intercalated cells B; PODO: podocytes; ENDO: endothelial cells; MES: mesangial cells; FIB: fibroblasts; LEUK: leukocytes; FR-PTC: failed-repair proximal tubule cells; TAL: thick ascending limb; DCT: distal convoluted tubule; CNT_PC: connecting tubule/principal cells.

We next explored the potential kidney origin of CCL20 by data mining kidney transcriptomics databases. In human DKD, CCL20 was found mainly expressed in an injured population of proximal tubular cells expressing VCAM (PTVCAM) and the tubular injury marker HAVCR1/KIM-1, as well as by parietal epithelial cells (PECs), which were at a much lower proportion and displayed negligible or barely detectable CCL20 expression in control human kidneys [20,21] (Figure 6A). In human ADPKD, compared to control kidneys, CCL20 was predominantly expressed by a major proportion of failed-repair proximal tubular cells (FR-PTC). CCL20 expression was also detected in the ascending thin limb (ATL), but only in injured kidneys and not in controls (Figure 6B) [20,22]. Overall, these findings are consistent with a kidney or tubular origin for urinary CCL20.

**Figure 7:**
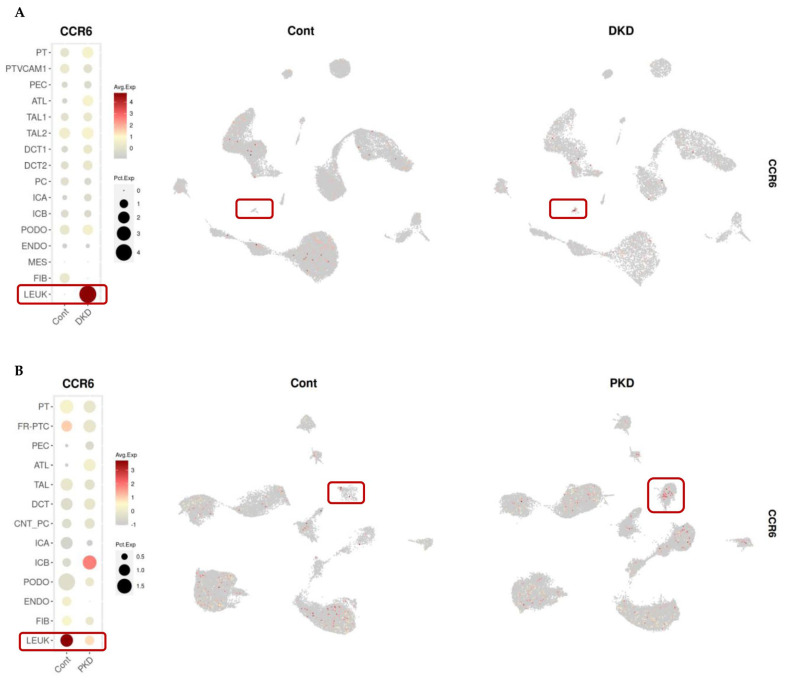
CCR6 expression in kidney cell populations from patients with DKD (**A**) and ADPKD (**B**). Data visualised using the KIT platform, developed by the Humphreys Lab (https://humphreyslab.com/SingleCell/) [20] based on snRNA-seq datasets from Wilson et al. (2019) and Muto et al. (2022), respectively [21,22]. The dot plots (left) indicate average expression (colour scale) and proportion of expressing cells (dot size) across kidney cell populations. UMAPs (right) illustrate the spatial distribution of CCL20-positive cells in single-nucleus RNA-seq datasets. Red boxes in the dot plots and UMAPs indicate the cell populations with CCR6 expression.

Locally, the main target of CCL20 expressed by proximal tubular cells in DKD and ADPKD appears to be CCR6-expressing leucocytes (Figure 7), in line with prior reports [23].

Furthermore, in agreement with KIT platform data, the transcriptomic data from Nephroseq confirmed increased CCL20 and CCR6 expression in human DKD samples compared to controls (Table 2).

## 3. Discussion

The results obtained in this study show high CCL20 levels in both patients with DKD and those with ADPKD, compared to the reference group. This suggests a possible involvement of the CCL20/CCR6 pathway in the pathophysiology of both diseases.

Although DKD and ADPKD are both causes of CKD, they differ in the systemic vs. local origin of the insult and in clinical trajectories. However, CCL20 levels were similar in both groups. Moreover, the lack of correlation between plasma and urinary CCL20 levels provides evidence for local kidney CCL20 production. This hypothesis was supported by data obtained through the Nephroseq and KIT database [20,21,22,24,25,26,27,28].

Taken together, these findings and the local expression of both CCL20 and its receptor CCR6 argue in favour of the potential role of CCL20 in kidney disease pathophysiology, although the precise role remains unclear, given the contradictory preclinical evidence in AKI resulting from different triggers [15,16] and the lack of association with long-term hard outcomes in the present cohorts treated according to clinical guidelines. While treatment according to guidelines may have silenced an association of baseline CCL20 levels with outcomes, the unmet clinical need is for biomarkers that allow risk stratification in patients who are optimally treated. This will allow us to select patients for future clinical trials of novel interventions.

The local production of CCL20 would be consistent with the lack of correlation between plasma and urinary CCL20 and may also be consistent with the trend towards higher plasma levels with more severe (larger kidneys) ADPKD, as well as higher urinary levels in DKD patients with A2–A3 albuminuria or in the G4 category. However, other sources of CCL20 should be hypothesised to account for the higher plasma CCL20 in early CKD in DKD, a systemic disease. A balance between cell stress triggering CCL20 expression and progressive loss of tubular cells (as the main source of CCL20) with CKD progression may preclude observing more clear-cut differences.

Overall, the present study displays data that may help to implement a precision medicine approach to the field of kidney disease [29,30]. CKD is among the fastest-growing global causes of death, projected to become the 5th global cause of death by 2050 [1]. More specifically, it is forecasted to become the third cause of death in countries with long life expectancy, such as Japan and Western Europe [31]. This has led to calls for research on biomarkers that identify the earliest stages of the disease, which may be termed pre-CKD in an analogy to prediabetes and pre-heart failure, two CKM conditions that share therapeutic approaches such as SGLT2I and glucagon-like peptide-1 receptor agonists (GLP1Ras) [32,33,34,35,36]. In this regard, ADPKD may be diagnosed based on imaging at a “pre-CKD” stage from the eGFR and albuminuria point of view, i.e., in the so-called blind-spot of CKD [37,38]. Thus, it is interesting that high plasma CCL20 levels were found in patients with ADPKD and eGFR G1–G2 with albuminuria A1, i.e., patients who may not be diagnosed with CKD based on eGFR and albuminuria. Additionally, high plasma and urinary CCL20 levels were observed in patients with DKD and preserved eGFR (G1–G2) or low albuminuria values (A1). The development of novel biomarkers (including inflammatory biomarkers, such as CCL20) and the use of artificial intelligence tools have been suggested for earlier detection of CKD, and redefinition of risk based on age-adapted eGFR values has been suggested to aid in this purpose [39,40,41,42]. CCL20 data may eventually be incorporated into these algorithms. While plasma levels alone were not informative for long-term outcomes, urinary levels appear to be related to early outcomes, yet not with long-term ones. The combination of CCL20 with other biomarkers and assessment of larger cohorts of early-stage disease may uncover its full potential as a risk stratification biomarker [43]. In this regard, in 331 haemodialysis patients, circulating CCL20 was independently associated with increased risk of all-cause or cardiovascular death and with cardiovascular events in a multivariable-adjusted model [44]. Additionally, in a large discovery sample (*n* = 1316, 249 with cardiovascular events), circulating CCL20 levels were associated with cardiovascular events, although this was not confirmed in a much smaller (71 events in 283 individuals) replication sample with shorter follow-up [45]. Finally, the CCL20-CCR6 axis is associated with several cancers, including kidney cancer [46]. Kidney cancer is part of the natural history of CKD, so longer-term studies should address a potential association with CCL20 in biological fluids [47].

Data mining evidence points to stressed proximal tubular cells as a key source of CCL20. This aligns well with the recently characterized role of proximal tubular cells as central hubs that integrate stress signals arising from tubular stress, glomerular injury, or hyperfiltration, thereby driving local proinflammatory and profibrotic responses. This has been demonstrated by spatial single-cell transcriptomics and by the therapeutic effects of drugs targeting proximal tubular cells, such as sodium–glucose cotransporter-2 inhibitors (SGLT2I) [23], fitting with accumulating evidence on their prominent role in CKD progression, derived from clinical trials of SGLT2I [48,49]. Overall, the data are consistent with current understanding of the regulation of CCL20 expression, especially under inflammatory conditions. In kidney cells, CCL20 is upregulated by classical proinflammatory mediators, such as nuclear factor kappa-light-chain-enhancer of activated B cells 1 (NF-κB1) and anaphylatoxins C3a and C5a [50,51]. In immune-mediated glomerulonephritis, response gene to complement-32 (RGC-32) promoted CCL20 expression [52]. In proximal tubular cells, activation of the cholinergic anti-inflammatory pathway by α7 nicotinic acetylcholine receptors (α7nAChRs) suppressed CCL20 production [53]. From a metabolic point of view, transcriptionally, CCL20 was one of four chemokines and chemokine receptor hubs identified as upregulated in DKD and downregulated in more advanced kidney failure [19]. In this regard, CCL20 is also under mitochondrial metabolic control, and its production is inhibited by inhibiting succinate dehydrogenase, which is a kidney protective intervention [54,55]. Succinate dehydrogenase (SDH) itself is a target of antidiabetic kidney protective drugs such as GLP1RA [56]. Interestingly, CCL20 activity in the kidney may be downregulated by locally expressed atypical chemokine receptors (ACKRs) that scavenge chemokines, such as ACKR3, ACKR4 and GPR182 [57]. Of these, proximal tubular cells express ACKR3 and mainly ACKR4 in both human DKD and ADPKD [21,22]. Among microRNAs (miRNAs), miR-143-5p downregulates CCL20 expression, and this is therapeutically relevant for kidney disease [58].

Some limitations should be acknowledged. The sample size was limited and may have precluded the detection of statistically significant differences that may become evident when analysing several hundred patients. The limited sample size did not allow multivariate models to explore how much prediction plasma cytokine levels add in combination with other factors associated with CKD progression. Moreover, this study was unicentric, so the results should be validated in a multicentre study. In this regard, there are limitations concerning the generalizability of the collected data.

This study also has strengths. Thus, patients were followed prospectively long-term at the nephrology clinic, beginning at the point of referral from primary care, and were provided with optimal care under real-world, routine care conditions, adjusted according to successive contemporary guidelines. Thus, the studies of association of cytokine levels with CKD progression or death do not reflect natural history but residual risk despite the fact that optimised treatment and progression rates were consistent with those reported for the intervention arm (SGLT2I, GLP1RAs, and mineralocorticoid receptor antagonists (MRAs)) of recent clinical trials in patients with DKD [59,60,61].

In conclusion, plasma and urinary CCL20 levels are high in two common forms of human CKD, one triggered by systemic events and the other by genetic defects, which have a different clinical course (early vs. later albuminuria, decreasing vs. increasing kidney size). These differences may have conditioned the observation that in DKD, higher CCL20 levels were observed in earlier stages of the disease, when the tubular cell mass was relatively preserved, and in ADPKD in later stages of the disease, associated with increased kidney size. This observation is consistent with data mining evidence of local CCL20 production in injured kidneys. This information provides insight into disease pathophysiology and is consistent with a potential role of CCL20 assessments for risk stratification in the clinic. Further studies are required in this regard, as in the context of the present study, their role in risk stratification appears to be limited.

## 4. Materials and Methods

### 4.1. Study Design and Study Population

**Figure A1:**
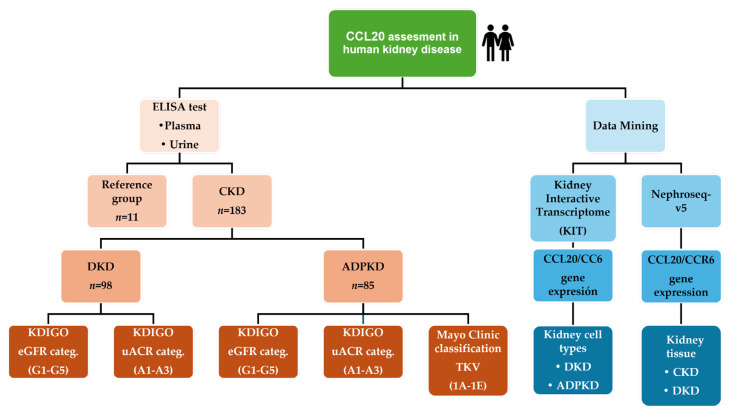
Flow chart of the study design to assess CCL20 in human kidney disease. CCL20 expression levels were analysed by ELISA in plasma and urine samples from CKD patients with diabetic kidney disease (DKD) or autosomal dominant polycystic kidney disease (ADPKD). Participants in each cohort were categorised according to eGFR and UACR criteria defined by KDIGO. ADPKD patients were further stratified using the Mayo Clinic total kidney volume (TKV) classification. In parallel, the origin and expression of CCL20 and its receptor CCR6 in DKD and ADPKD were explored using publicly available transcriptomic resources, Kidney Interactive Transcriptome (KIT) and Nephroseq, to assess their transcriptional expression in individual kidney cell types and whole/microdissected kidney tissue, respectively.

**Figure A2:**
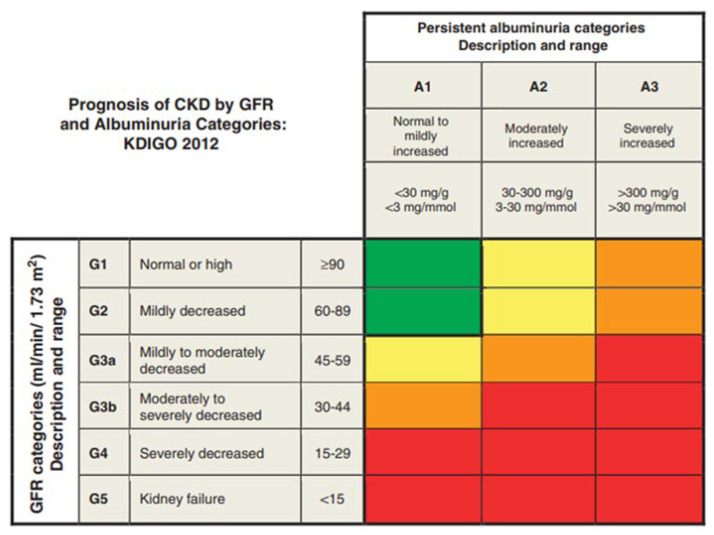
KDIGO risk categories of CKD according to eGFR (G) and albuminuria (A) categories [63]. Green: low risk (if no other markers of kidney disease, no CKD); yellow: moderately increased risk; orange: high risk; red: very high risk.

This is an observational, cross-sectional study with prospective follow-up of 98 incident adults with DKD and 85 adults with ADPKD under real-world, routine care conditions in a monographic outpatient nephrology clinic of a tertiary hospital who had biobanked biosamples. CKD albuminuria (A) and GFR (G) categories were defined according to KDIGO guidelines on CKD from 2012 and updated in 2024 [62,63]. In patients with ADPKD, additional risk stratification was performed according to the Mayo Clinic imaging classification based on total kidney volume (TKV) [64]. Additionally, samples from 11 healthy subjects without kidney disease were studied as a reference group (Figure A1). KDIGO risk categories were assigned based on eGFR and UACR values (Figure A2).

Patients were spontaneously referred to nephrology by primary care physicians or other specialists, and referred patients were consecutively enrolled if they consented. The protocol was approved by the IIS-Fundacion Jimenez Diaz Ethics Committee (EO030-20 and EO297-24). Participants signed an informed consent form before enrolment.

**Figure A3:**
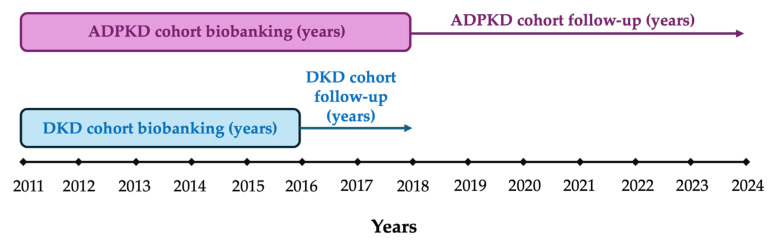
Study flow diagram illustrating the biobanking and follow-up periods for participants in the ADPKD and DKD cohorts. Coloured bars indicate the duration of sample collection (biobanking), while arrows represent ongoing clinical follow-up.

Adults with a clinical diagnosis of DKD referred to the nephrology department between July 2011 and June 2016, and those with a clinical diagnosis of ADPKD referred between June 2014 and November 2016, were included in this study if biobanked urine and/or blood samples were available. DKD patients were followed until December 2018, and ADPKD patients until December 2024 (Figure A3). During follow-up, care was adapted according to evolving American Diabetes Association (ADA) and Improving Global Outcomes (KDIGO) clinical practice guidelines in a real-world, routine clinical practice setting, along with the appropriate care for ADPKD [65,66,67,68,69]. All patients underwent assessment of treatments, as well as blood and urine tests. Exclusion criteria included age under 18 years, KRT, positive serology for hepatitis B virus (HBV), hepatitis C virus (HCV), or human immunodeficiency virus (HIV), and unwillingness to participate.

### 4.2. CCL20 Assay

Plasma and urinary CCL20 were determined by enzyme-linked immunosorbent assay (ELISA, DY360; R&D Systems, Minneapolis, MN, USA), as previously reported [15]. When CCL20 was undetectable, samples were concentrated fourfold using Amicon Ultra 3K centrifugal filter units (Merck Millipore Ltd., Carrigtwohill, County Cork, Ireland). Samples that remained undetectable for CCL20, even after concentration, were considered negative for CCL20 and assigned a value of 0.0 pg/mL (LOD = 2.0 pg/mL) to enable their inclusion in the comparative analysis.

### 4.3. Data Mining

Data mining for CCL20 in human CKD transcriptomics datasets was performed using Nephroseq v5 (http://v5.nephroseq.org/, accessed on 26 October 2025) [24,25,26,27,28]. A high-sensitivity approach was used, in which statistically significant differences (*p* < 0.05) in gene expression or correlation with analytical values were selected. Kidney cell types expressing CCL20 and its receptor CCR6 were explored using Kidney Interactive Transcriptomics in human DKD and ADPKD single-cell transcriptomics datasets [20,21,22,70].

### 4.4. Statistical Analysis

Experimental results were statistically analysed using GraphPad Prism version 9. Data are presented as medians and interquartile ranges (IQR) unless otherwise specified. Normality was assessed using the Kolmogorov–Smirnov test. Group comparisons were performed using the Mann–Whitney U test or the Kruskal–Wallis test, depending on whether the comparison was binary or involved multiple groups, respectively. Contingency tables and Fisher’s exact test were used to compare CCL20 detection between patients and the reference group, as well as across different clinical categories of disease. Correlation analyses were performed using the Spearman correlation coefficient. Time to death or initiation of kidney replacement therapy in patients diagnosed with DKD and ADPKD was analysed using Kaplan–Meier survival curves, compared with the log-rank test, and corresponding hazard ratios (HR) were calculated. A two-sided *p*-value < 0.05 was considered statistically significant. Statistical results from Nephroseq are presented as reported on the official website.

## Figures and Tables

**Table 1:** Baseline characteristics and outcome of patients with diabetic kidney disease and autosomal dominant polycystic kidney disease.

	Age (Years)	Male, n (%)	eGFR (mL/min/1.73 m^2^)	UACR (mg/g)	TKV (mL)	KRT at Baseline *n* (%)	KRT, *n* (%)	Death, *n* (%)	Follow-Up Time (Years)
DKD *n* = 98	69.1 (60.4–76.4)	75 (76.5)	57.6 (70.7–83.2)	159.1 (36.9–437.7)	NA	0(0)	5 (5.1)	21 (21.4)	4.9 (2.9–5.5)
ADPKD *n* = 85	54.75 (43.2–67.6)	39 (45.9)	59.26 (37.5–95.6)	25.35 (7.1–77.3)	1526 (667.8–2586)	10 (11.8)	10 (11.8)	28 (32.9)	7.1 (2.9–8.4)

Median values are shown with interquartile range (IQR). G1–G5: chronic kidney disease (CKD) stages based on estimated glomerular filtration rate (eGFR). A1–A3: albuminuria categories based on urinary albumin-to-creatinine ratio (UACR). 1A–1F: Mayo Clinic categories based on height-adjusted total kidney volume (TKV). Kidney replacement therapy (KRT) at baseline indicates patients already receiving kidney replacement therapy at study entry. Patients who initiated KRT shortly before death were not classified as KRT-treated. Data shown are from patients with diabetic kidney disease (DKD) and autosomal dominant polycystic kidney disease (ADPKD). NA: not available.

**Table 2:** Data mining: bulk human kidney transcriptomics from the Nephroseq database [24]. The table shows differential expression levels of CCL20 and CCR6 in whole kidney or glomerular (Glom) or tubulointerstitial (TubInt) compartments, expressed as fold change and corresponding *p*-values.

Gene	Dataset	Disease	*n*	Fold Change	*p*-Value	References
*CCL20*	Nakagawa CKD Kidney	CKD vs. Normal Kidney (Discovery set)	53	3.9	1.27 × 10^−7^	25
	Nakagawa CKD Kidney	CKD vs. Normal Kidney (Validation set)	8	12.4	0.001	25
	Woroniecka DKD TubInt	DKD vs. Healthy Living Donor	22	2.1	0.006	26
	Ju CKD Glom	DKD vs. Healthy Living Donor	33	2.1	0.008	27
	Schmid DKD TubInt	Nephrotic vs. Subnephrotic (DKD)	11	1.6	0.01	28
*CCR6*	Nakagawa CKD Kidney	CKD vs. Normal Kidney (Discovery set)	53	2.2	0.001	25
	Nakagawa CKD Kidney	CKD vs. Normal Kidney (Validation set)	8	5.1	0.012	25

## Data Availability

Data from the Kidney Interactive Transcriptomics (KIT) and Nephroseq databases presented in this study were derived from the following resources available in the public domains: https://humphreyslab.com/SingleCell/ and https://www.nephroseq.org/resource/login.html (accessed on 26 October 2025).

## Abbreviations

The following abbreviations are used in this manuscript:

CKD	Chronic kidney disease
CKM	Cardiovascular–metabolic–kidney spectrum
CAKUT	Congenital anomalies of the kidney and urinary tract
ADPKD	Autosomal dominant polycystic kidney disease
CCL20	CC motif chemokine ligand 20
CCR6	CC chemokine receptor type 6
Th17 cells	T helper 17 cells
Treg cells	Regulatory T cells
AKI	Acute kidney injury
DKD	Diabetic kidney disease
IQR	Interquartile range
eGFR	Estimated glomerular filtration rate
UACR	Urine albumin-to-creatinine ratio
KRT	Kidney replacement therapy
TKV	Total kidney volume
Ref	Reference group
ELISA	Enzyme-linked immunosorbent assay
ns	Not significant
HR	Hazard ratio
PTVCAM	Proximal tubular cells expressing VCAM
PEC	Parietal epithelial cells
FR-PTC	Failed-repair proximal tubular cells
ATL	Ascending thin limb
KIT	Kidney Interactive Transcriptomic
snRNA-seq	Single-nucleus RNA sequencing
UAMPs	Uniform Manifold Approximation and Projection
Cont	Control
PT	Proximal tubule
TAL1	Thick ascending limb 1
TAL2	Thick ascending limb 2
DCT1	Distal convoluted tubule 1
DCT2	Distal convoluted tubule 2
PC	Principal cells
ICA	Intercalated cells A
ICB	Intercalated cells B
PODO	Podocytes
ENDO	Endothelial cells
MES	Mesangial cells
FIB	Fibroblasts
LEUK	Leukocytes
TAL	Thick ascending limb
DCT	Distal convoluted tubule
CNT_PC	Connecting tubule/Principal cells
SGLT2I	Sodium-glucose cotransporter-2-inhibitor
Glom	Glomerular
TubInt	Tubulointerstitial
GLP1Ras	Glucagon-like peptide-1 receptor agonist
NF-κB1	Nuclear Factor kappa-light-chain-enhancer of activated B cells 1
RGC-32	Response gene to complement-32
α7nAChRs	α7 nicotinic acetylcholine receptors
SDH	Succinate Dehydrogenase
ACKRs	Atypical chemokine receptors
miRNAs	MicroRNAs
MRAs	Mineralocorticoid receptor antagonists
ADA	American Diabetes Association
KDIGO	Kidney Disease: Improving Global Outcomes
HBV	Hepatitis B virus
HCV	Hepatitis C virus
HIV	Human immunodeficiency virus
LOD	Limit of detection

## Appendix A. Supplementary Baseline Characteristics

This appendix provides additional details on the baseline characteristics of study participants.

### Appendix A.1. Detailed Characteristics and Outcomes of Patients with Diabetic Kidney Disease

Table A1 shows the clinical characteristics of patients with DKD, stratified by eGFR and UACR.

**Table A1:** Baseline characteristics and outcome of patients with DKD.

		*n* (%)	Age (Years), Median (IQR)	Male, *n* (%)	eGFR (mL/min/1.73 m^2^), Median (IQR)	UACR (mg/g), Median (IQR)	KRT, *n* (%)	Death, *n* (%)	Follow-Up (Years), Median (IQR)
Patients		98 (100)	69.1 (60.4–76.5)	75 (76.5)	57.6 (70.7–83.2)	159.1 (36.9–437.7)	5 (5.1)	21 (21.4)	4.9 (2.9–5.5)
eGFR categories	G1	18 (18.4)	57.8 (51.7–68.2)	16 (88.9)	95.4 (92.23–98.08)	311.1 (63.4–517.0)	0 (0)	2 (11.1)	4.9 (3.0–5.7)
	G2	26 (26.5)	67.1 (58.2–71.6)	19 (73.1)	75.7 (69.4–83.2)	154.8 (45.3–313.2)	0 (0)	5 (19.2)	4.2 (2.6–5.4)
	G3	42 (42.9)	71.6 (64.7–7.6)	32 (76.2)	45.6 (39.9–55.0)	77.3 (24.6–413.2)	1 (2.4)	9 (21.4)	5.0 (3.6–5.4)
	G4	11 (11.2)	76.4 (63.5–84.1)	7 (63.6)	25.9 (22.2–28.6)	312.4 (7.4–823.0)	4 (36.4)	4 (36.4)	4.8 (1.9–5.9)
	G5	1 (1.0)	61.7	1 (100)	10.7	430.9	0(0)	1 (100)	4.1
UACR categories	A1	22 (22.5)	74.6 (64.8–8.1)	16 (72.7)	44.7 (32.3–64.4)	12.7 (6.1–16.85)	0 (0)	7 (31.8)	5.0 (2.2–5.6)
	A2	39 (39.8)	66.5 (59.9–73.2)	30 (76.9)	72.1 (46.8–83.9)	78.1 (55.5–181.8)	1 (2.6)	1 (2.6)	5.0 (3.3–5.6)
	A3	37 (37.8)	68 (60.3–77.4)	29 (78.4)	57.0 (35.7–90.0)	554.7 (371.9–974.2)	4 (10.8)	13 (35.1)	4.2 (2.8–5.4)

Median values are presented with interquartile range (IQR). G1–G5: CKD stages based on eGFR. A1–A3: albuminuria categories based on UACR. Patients who initiated KRT shortly before death were not counted as KRT-treated.

### Appendix A.2. Detailed Characteristics and Outcomes of Patients with Autosomal Dominant Polycystic Kidney Disease

Table A2 shows the clinical characteristics of patients with ADPKD, stratified by eGFR, UACR and Mayo Clinic TKV classification.

**Table A2:** Baseline characteristics and outcome of patients with ADPKD.

		*n* (%)	Age (Years), Median (IQR)	Male, *n* (%)	eGFR (mL/min/1.73 m^2^), Median (IQR)	UACR (mg/g), Median (IQR)	KRT, *n* (%)	Death, *n* (%)	Follow-Up (Years), Median (IQR)
Patients		85 (100)	54.8 (43.2–67.6)	39 (45.9)	59.3 (37.5–95.6)	25.4 (7.1–77.3)	10 (11.8)	28 (32.9)	7.1 (2.9–8.4)
eGFR categories	G1	22 (25.9)	36.6 (32.0–44.5)	8 (36.4)	104.3 (98.6–115.4)	8.3 (3.7–19.1)	0 (0)	6 (27.3)	7.4 (2.2–8.3)
	G2	20 (23.5)	58.9 (49.9–73.2)	9 (45)	70.3 (64.6–82.2)	20.3 (6.9–62.0)	0 (0)	4 (20)	7.9 (6.4–8.6)
	G3	29 (34.1)	65.1 (54.6–73.9)	13 (44.8)	44.0 (37.5–57.5)	27.5 (7.1–94.3)	1 (3.5)	14 (48.3)	7.2 (4.7–8.5)
	G4	8 (9.4)	57.3 (44.8–68.0)	6 (75)	25.0 (22.3–26.8)	80.3 (51.4–163.6)	5 (62.5)	3 (37.5)	2.9 (1.5–3.1)
	G5	6 (7.1)	50.6 (43.0–69.5)	3 (50)	11.0 (8.3–12.3)	193.2 (68.1–269.5)	4 (66.7)	1 (16.7)	1.5 (0.3–3.9)
UACR categories	A1	45 (52.9)	51.9 (37.4–63.8)	19 (42.2)	81.2 (58.0–103.1)	7.5 (3.3–14.7)	0 (0)	9 (20)	7.7 (6.1–8.5)
	A2	33 (38.8)	60.4 (45.9–72.0)	13 (39.4)	38.0 (25.0–69.1)	69.0 (51.3–139.5)	8 (24.2)	14 (42.4)	4.7 (2.6–8.0)
	A3	6 (7.1)	63.2 (55.4–77.4)	6 (100)	43.5 (23.3–64.1)	713.5 (320.0–958.3)	1 (10)	5 (90)	1.8 (0.3–4.4)
TKV categories	1A	12 (14.1)	56.3 (38.6–66.7)	0 (0)	74.3 (42.0–105.3)	6.0 (2.2–58.6)	1 (8.3)	3 (25)	6.5 (2.3–7.5)
	1B	14 (16.5)	66.1 (49.0–73.9)	8 (57.1)	77.8 (42.0–102.3)	12.1 (6.2–39.2)	0 (0)	5 (35.7)	7.4 (4.9–8.4)
	1C	23 (27.1)	55.2 (43.7–66.4)	13 (56.5)	59.3 (33.0–88.4)	21,3 (6.3–57.9)	2 (8.7)	5 (21.7)	7.6 (3.0–8.5)
	1D	11 (12.9)	53.4 (41.1–57.5)	4 (36.4)	52.0 (27.0–83.1)	10.0 (6.4–69.0)	2 (18.2)	2 (18.2)	7.6 (3.11–8.3)
	1E	6 (7.1)	43.7 (38.3–48.0)	5 (90)	27.5 (19.5–49.3)	108.2 (37.5–199.6)	4 (66.7)	2 (33.3)	2.5 (1.5–3.9)

Median values are presented with interquartile range (IQR). G1–G5: CKD stages based on estimated glomerular filtration rate (eGFR). A1–A3: albuminuria categories based on urinary albumin-to-creatinine ratio (UACR). 1A–1E: Mayo Clinic categories based on height-adjusted total kidney volume (TKV). Patients who initiated KRT shortly before death were not classified as KRT-treated.
